# Multifocal anaplastic astrocytoma mimicking primary central nervous system lymphoma: A case report

**DOI:** 10.1097/MD.0000000000047043

**Published:** 2026-01-02

**Authors:** Bufan Yang, Hongyuan Liu, Guoliang You, Shiquan Wen, Yufeng Tang

**Affiliations:** a Department of Neurology, Mianyang Central Hospital, School of Medicine, University of Electronic Science and Technology of China, Mianyang, Sichuan Province, People’s Republic of China; b Department of Neurosurgery, Mianyang Central Hospital, School of Medicine, University of Electronic Science and Technology of China, Mianyang, Sichuan Province, People’s Republic of China; c Department of Neurosurgery, Mianyang People’s Hospital, Mianyang, Sichuan Province, People’s Republic of China.

**Keywords:** craniotomy, misdiagnosis, multifocal anaplastic astrocytoma, primary central nervous system lymphoma, stereotactic biopsy

## Abstract

**Background:**

Multifocal anaplastic astrocytoma (MAA) is exceedingly rare. The atypical clinical symptoms and imaging characteristics pose significant challenges for accurate clinical diagnosis.

**Case presentation:**

A 64-year-old woman presented with clinical symptoms persisting for 3 months, characterized by left-sided facial numbness, an unsteady gait that was lateralized, progressive worsening of incomplete closure of the left eye, dysphagia, and episodes of choking. Based on her clinical symptoms and magnetic resonance imaging (MRI), she was misdiagnosed as primary central nervous system lymphoma. Three months later, her clinical symptoms worsened, and she was admitted to neurosurgery where she underwent a stereotactic biopsy of the left cerebellar hemisphere. Histopathological examination and immunohistochemical analysis were consistent with anaplastic astrocytoma, IDH wild-type status, classified as World Health Organization grade 3.

**Conclusion:**

MAA exhibits a rapid progression and is associated with a poor prognosis. Clinicians must enhance their understanding of the MRI manifestations of MAA. Moreover, when MRI findings are insufficient to establish a definitive diagnosis, brain stereotactic examination becomes essential.

## 1. Introduction

Glioma represents a heterogeneous group of tumors, with anaplastic astrocytoma (AA) accounting for 4% of all malignant central nervous system tumors and 10% of all gliomas.^[1]^ AA predominantly occur in the brain, followed by the cerebellum. However, AA located in the brainstem constitutes only 3.6% of cases.^[2]^ The clinical manifestations observed in patients with AA are diverse and primarily depend on tumor location. These may include focal neurological deficits, headaches, speech disorders, dementia, gait disturbances, and epileptic seizures. Typically, magnetic resonance imaging (MRI) findings for AA reveal low signal intensity on T1-weighted imaging (T1WI) and high signal intensity on T2-weighted imaging (T2WI) accompanied by surrounding vascular edema. Nodular enhancement – often indicative of high-grade components is frequently noted. However, it is important to recognize that approximately one-third of AAs do not exhibit contrast enhancement.^[3]^ Multifocal anaplastic astrocytoma (MAA) is an even rarer entity, sparsely documented in the literature. MAA is characterized by the presence of multiple tumor foci within the cerebral parenchyma, posing significant diagnostic and therapeutic challenges due to its genetic complexity, overlapping clinical presentations with other gliomas, and universally unfavorable prognosis. Herein we report a case of MAA characterized by progressive symptoms that were misdiagnosed as primary central nervous system lymphoma (PCNSL) over a period of 3 months.

## 2. Case presentation

**Figure 1.**
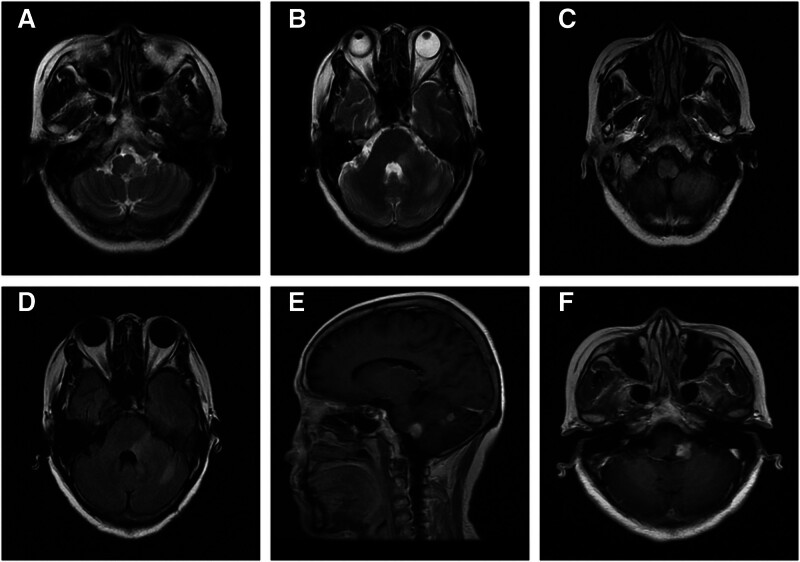
Initial admission cranial MRI. The left pons and cerebellar hemispheres exhibited slightly elevated lesion signals on both T1WI and T2WI (A–D). The enhanced MRI of the head revealed nodular enhancement of the lesions located in the left pontine region and cerebellum (E, F). MRI = magnetic resonance imaging, T1WI = T1-weighted imaging, T2WI = T2-weighted imaging.

A 64-year-old woman presented to the neurology department with a complaint of dizziness persisting for 20 days. The patient experienced persistent dizziness accompanied by unsteady gait. Additionally, there were intermittent episodes of choking and dysphagia following water intake, which were partially alleviated with rest. Upon admission, her vital signs were recorded: temperature at 36.8°C, heart rate at 74 beats/min, and blood pressure at 134/80 mm Hg. A neurological examination revealed no focal neurological deficits. There was no family history or disease in the past. Serum test results indicated elevated triglyceride levels at 7.75 mmol/L and low-density lipoprotein levels at 3.87 mmol/L, carcinoembryonic antigen, alpha-fetoprotein and thyroid hormone levels, complete blood cell count, blood glucose level, liver function tests, kidney function tests, and coagulation profile all normal results. Cerebrospinal fluid analysis via lumbar puncture showed colorless and transparent fluid with an opening pressure of 120 mm H_2_O, red blood cell counts and white blood cell counts including lymphocytes and monocytes remained within normal ranges. The glucose level in cerebrospinal fluid was measured at 4.4 mmol/L while chloride ion concentration was found to be 125.5 mmol/L, protein concentration was noted to be low at 0.48 g/L. Testing for antibodies against aquaporin-4 (cerebrospinal fluid water channel protein-4), oligodendrocyte glycoprotein associated with myelin sheath integrity, as well as myelin phospholipid alkaline protein yielded negative results. Transcranial Doppler ultrasound examination did not reveal any abnormalities. Three-dimensional computed tomography imaging of the intracranial arteries demonstrated that the right vertebral artery appeared slender while the left dominant vertebral artery provided adequate perfusion without significant abnormalities detected in other vessels examined. The left pons and cerebellar hemispheres exhibited slightly elevated lesion signals on both T1WI and T2WI (Fig. 1A–D). Enhanced MRI of the head revealed nodular enhancement of the lesions located in the left pontine region and cerebellum (Fig. 1E, F). Before undergoing magnetic resonance spectroscopy (MRS), the patient requested discharge from the hospital. After discharge, the patient underwent MRS at another hospital. MRS demonstrated an elevated choline, decreased *N*-acetylaspartate, and increased choline-to-creatinine. The patient’s clinical presentation and ancillary investigations were consistent with a diagnosis of PCNSL. The patient declined pharmacotherapeutic intervention.

**Figure 2.**
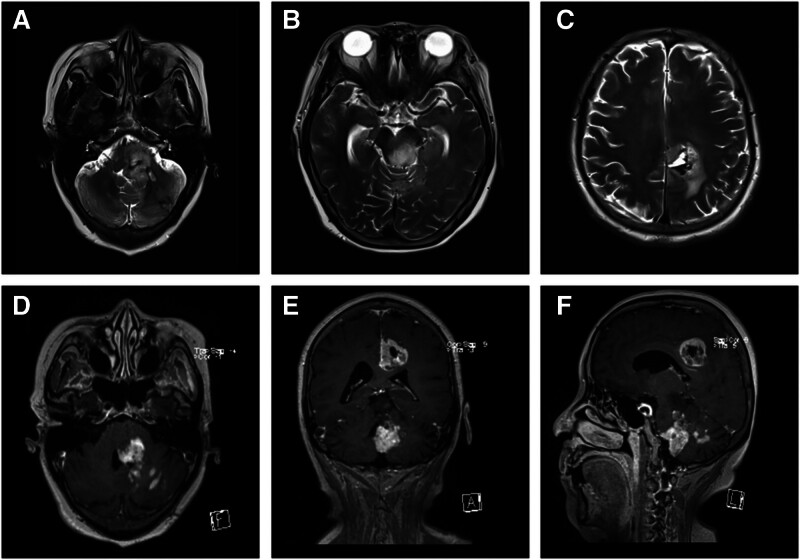
The second cranial MRI examination demonstrated an increase in both the size and diffusion of the occupying lesion, accompanied by significant enhancement. The cranial MRI of the patient revealed multiple space-occupying lesions located in the midline region of the brain. T2WI (A–C) and enhanced MRI (D–F) demonstrated high-signal space-occupying lesions in the left cerebellum and brainstem adjacent to the fourth ventricle. T2WI indicated a high-signal mass within the pons (A, B). Additionally, T2WI (C) exhibited an irregular high-signal mass in the left parietal lobe. Coronal enhanced MRI displayed an irregular high signal in both the left cerebellum and left parietal lobe near the lateral ventricle (E). Sagittal enhanced MRI further illustrated irregular high signals present in the brainstem, fourth ventricle, cerebellum, and parietal lobe (F). MRI = magnetic resonance imaging, T1WI = T1-weighted imaging, T2WI = T2-weighted imaging.

The patient’s condition deteriorated after 3 months, leading to admission to the neurosurgery department. The patient presented with exacerbating dizziness, left-sided limb weakness accompanied by an unsteady gait, sensory impairment on the left side of the face, difficulty in closing the left eye, blurred vision, diplopia, dysphagia, and increased coughing while drinking water. MRI of the head revealed that the lesion involving the left pons and left cerebellar hemispheres had enlarged compared to previous assessments, extending superiorly into the left parietal lobe. Diffusion-weighted imaging and T2WI demonstrated slightly elevated signal intensities. Contrast-enhanced MRI indicated nodular enhancement of the lesion with irregular margins (Fig. 2D–F). Taking into account the patient’s clinical symptoms, medical history, and findings from MRI collectively shifted diagnostic suspicion from PCNSL to glioma.

**Figure 3.**
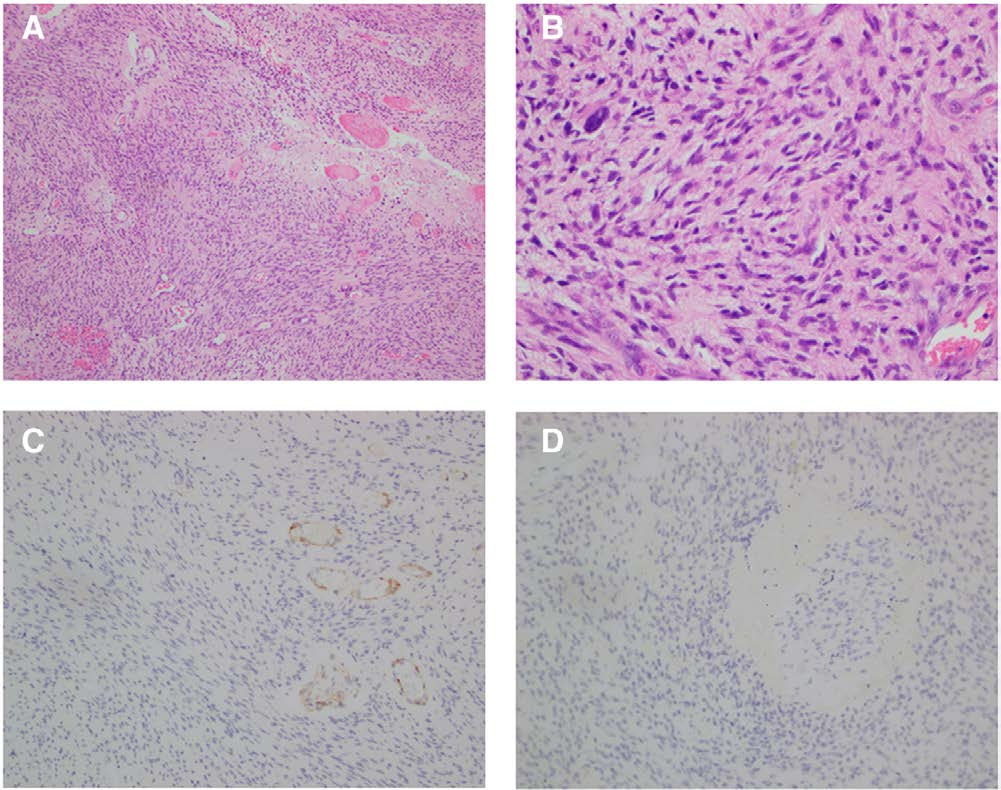
Immunohistochemical and pathological analysis of lesions acquired via stereotactic puncture. Hematoxylin and eosin staining of this lesion reveals that tumor cells are distributed in patches, exhibiting mitotic figures, a dense cellular arrangement, and mild to moderate atypia (A, B). Immunohistochemical staining indicates that the tumor cells are negative for IDH1 and H3K27M (C, D).

The patient declined craniotomy for resection of the intracranial lesion. A stereotactic biopsy was conducted on the lesion located in the left cerebellar hemisphere. The histopathological characteristics and immunohistochemical findings were consistent with AA, IDH wild-type, classified as World Health Organization grade 3 (Fig. 3). The patient refused chemotherapy and radiotherapy after the surgery, and been treated with long-term oral Chinese medicine. Unfortunately, we received the distressing news that the patient passed away more than 6 months after diagnosis.

## 3. Discussion

Multifocal cerebral glioma (MCG) accounts for approximately 10% of gliomas, and its pathogenesis remains unknown. Current theories on MCG pathogenesis focus on 2 hypotheses: tumor cell dissemination via cerebrospinal fluid (CSF) or white matter tracts as carriers, and acquired or hereditary genetic defects “triggering” neoplastic transformation. Gururangan et al reported a case of MAA in a patient with multiple comorbidities, including hereditary colorectal cancer and PMS2 gene mutation.^[4]^ Felicella et al explored the genetic landscape of MAA, identifying a common amplification of the MYC gene on chromosome 8q in 2 distinct tumor sites within a patient. These cases highlight potential genetic predispositions and monoclonal origins underlying multifocal tumor development.^[5]^ In our case, lesions were predominantly located in the cerebellum, brainstem, and parietal lobe near the corpus callosum—regions associated with CSF flow. This observation supports the hypothesis that glioma cells may disseminate via CSF or white matter tracts. Further genetic validation is required.

Glioblastoma multiforme is the most common subtype in MCG, while MAA is exceedingly rare. MAA represents a complex and challenging subset of gliomas, characterized by multiple tumor foci within the brain. This condition is typically associated with poor prognosis and poses unique diagnostic and therapeutic challenges. MAA is often misdiagnosed as metastatic disease or other neurological disorders. Clinical manifestations vary depending on tumor location, including focal neurological deficits, headaches, speech disorders, dementia, gait disturbances, and epileptic seizures. MRI findings include hypointensity on T1WI, hyperintensity on T2WI with peritumoral edema, and heterogeneous contrast enhancement. MRS reveals elevated choline, reduced *N*-acetylaspartate, and increased choline-to-creatinine ratios. Kong et al described a case of MAA misdiagnosed as metastatic disease.^[6]^ Kantorová et al emphasized the diagnostic challenges posed by overlapping symptoms between MAA and conditions such as multiple sclerosis or progressive multifocal leukoencephalopathy.^[7]^ In our case, the patient’s initial MRI and MRS findings led to a misdiagnosis of PCNSL, underscoring the difficulty in distinguishing these pathologies and the risk of delayed treatment.

Histopathological and immunohistochemical features are critical for tumor classification. Microscopically, AA exhibits densely packed sheets of tumor cells with mild to moderate atypia and occasional mitotic figures. Immunohistochemical analysis in our case demonstrated GFAP (+), Olig2 (+), Ki-67 hot spots of 20%, ATRX (+), and faint p53 positivity. Molecular profiling revealed IDH1/2(−), H3K27M(−), H3K27me3(−), EZHIP(−), and no 1p/19q co-deletion. These findings align with a diagnosis of AA, IDH-wildtype, World Health Organization grade 3.

Prognosis for multifocal high-grade gliomas, including anaplastic astrocytoma, remains dismal, with median overall survival reported as low as 8 months.^[8]^ Current management of MAA involves a combination of surgical resection, radiotherapy, and chemotherapy, though efficacy varies significantly depending on molecular characteristics and the extent of multifocality.^[8]^ Wallner et al discussed patterns of treatment failure in MAA, providing insights into prognosis and therapeutic strategies. Understanding typical failure modes is essential for optimizing treatment and anticipating complications.^[9]^ In this case, the patient declined craniotomy, chemotherapy and radiotherapy, ultimately succumbing to the disease 6 months post-stereotactic biopsy.

## 4. Conclusion

MAA is a significant clinical entity, exceedingly rare, posing considerable challenges in diagnosis and treatment. Ongoing research is crucial to better understand the unique aspects of MAA and to improve patient outcomes.

## Acknowledgments

We would like to express our gratitude to the patient’s family for giving oral and written informed consent for the reporting and publication of the case.

## Author contributions

**Formal analysis:** Hongyuan Liu, Guoliang You.

**Investigation:** Bufan Yang, Hongyuan Liu, Guoliang You, Shiquan Wen.

**Methodology:** Bufan Yang.

**Resources:** Bufan Yang.

**Writing – original draft:** Bufan Yang, Hongyuan Liu.

**Writing – review & editing:** Bufan Yang, Yufeng Tang.
